# Sociodemographic disparities in awareness of chronic conditions: an observational study among older persons in rural north-east of South Africa

**DOI:** 10.1136/bmjph-2023-000315

**Published:** 2024-03-13

**Authors:** Daniel Ohene-Kwofie, Carlos Riumallo-Herl, Chodziwadziwa Kabudula, Francesc Xavier Gómez-Olivé

**Affiliations:** 1 MRC/Wits Rural Public Health and Health Transitions Research Unit (Agincourt), School of Public Health, University of the Witwatersrand, Johannesburg, Gauteng, South Africa; 2 Applied Economics, Erasmus Universiteit Rotterdam, Rotterdam, The Netherlands

**Keywords:** Public Health, HIV, Education, Medical

## Abstract

**Background:**

The prevalence of chronic diseases is high among the older population. Awareness of these conditions is a crucial prerequisite to initiate treatment, control and prevent further complications. This study evaluates sociodemographic disparities in awareness of chronic diseases among people 40 years and over in rural South Africa.

**Methods:**

Data from the baseline survey of the Health and Aging in Africa: A Longitudinal Study of an INDEPTH Community in South Africa were analysed to estimate the level of awareness of chronic conditions such as HIV, hypertension, diabetes and dyslipidaemia among the population 40+ years. We compare self-reported awareness with objective measurements and conduct logistic regressions to evaluate sociodemographic determinants of awareness of chronic conditions.

**Results:**

We find that 80% of individuals have at least one chronic condition—HIV, diabetes, hypertension and/or dyslipidaemia. Awareness rates were relatively high among those with at least one chronic condition but varied across conditions and genders: HIV (83% for women, 84% for men), hypertension (88% of women, 81% of men); diabetes (76% for women, 75% for men); dyslipidaemia (10% for both women and men). We observe differences across individual, household and community factors. Generally, women are more aware of their condition; awareness increases with age except for HIV; persons from high consumption per capita households, living with one or more persons and living closer to a health facility are more likely to be aware of their conditions.

**Conclusions:**

Older adults in rural South Africa are generally aware of their chronic conditions, though there are important differences by age, gender and socioeconomic status. However, there is still a fraction unaware of their conditions and, therefore, lacking the necessary information to initiate treatment and implement behavioural changes to control them. Our findings may guide policy-makers directing the required efforts to promote targeted awareness campaigns by sociodemographic/socioeconomic subgroups.

**WHAT IS ALREADY KNOWN ON THIS TOPIC**

- Awareness of chronic conditions has been studied in high-income settings identifying gender, income and education as important determinants of awareness. However, there is little evidence on the determinants of awareness in rural settings of low-income and middle-income countries where population are ageing rapidly.

**WHAT THIS STUDY ADDS**

- We find that awareness in a low-income rural setting is high but varies by condition; with 83% being aware of HIV, 85% of hypertension, 77% of diabetes and just 10% of dyslipidaemia.
- Age, gender and socioeconomic status are important determinants of chronic condition awareness in rural South Africa.

**HOW THIS STUDY MIGHT AFFECT RESEARCH, PRACTICE OR POLICY**

- At the policy and practice level, targeted efforts are required to increase awareness of chronic conditions for particularly vulnerable population.
- Improved screening efforts are required at the primary healthcare facilities, particularly, for dyslipidaemia which is an established and important risk factor for cardiovascular diseases.

## Introduction

 A United Nations report on population ageing has shown that the global population of older persons (aged 60+) is growing considerably faster than the general population.^1^ This is a consequence of the rapid decline in communicable diseases and greater survival to older ages. While the ageing population will increase worldwide between 2019 and 2050, the largest increases are projected to occur in Asia and Africa. In particular, sub-Saharan Africa is expected to have the second-largest increase in populations of older persons going from 32 million in 2019 to over 101 million by 2050.^1^

Population ageing is a global health challenge as older individuals are more likely to suffer from non-communicable chronic diseases (NCDs).^2^ These are caused by biological degeneration in the later period of human life.^3^,^7^ Hypertension, diabetes mellitus, chronic kidney disease and arthritis are among the leading ten degenerative diseases causing a reduced quality of life.^2 8^ Furthermore, according to the WHO, NCDs accounted for 74% of all deaths globally in 2019.^2 8^

As NCDs continue to expand, it is likely that national healthcare systems will become burdened as individuals with chronic conditions usually have larger consumption of medical and health resources.^9^ It is, therefore, urgent to implement effective intervention strategies to control the NCD epidemic. However, any successful intervention hinges on individual awareness of their condition to either prevent further deteriorations or for an effective self-management.

Existing studies have shown that patients’ better knowledge about their chronic condition, such as diabetes/hypertension, was associated with better medication adherence and better disease management.^9^,^14^ Despite many studies investigating chronic conditions and their determinants elsewhere, few have focused on the knowledge and awareness of chronic conditions among older populations and the sociodemographic factors associated with awareness in rural sub-Saharan Africa. Gómez-Olivé *et al*^15^ explored the regional and sex differences in the prevalence and awareness of hypertension as part of a Human Heredity and Health in Africa (H3Africa) study across six sites in sub-Saharan Africa including rural South Africa. The study found a high prevalence of hypertension, with 63.1% awareness in Agincourt.^15^ More so, Reiger *et al* identified a very low awareness and treatment of dyslipidaemia in the cohort.^16^ However, unlike previous studies on awareness in the region, we focus on the awareness of the older population across four different chronic conditions and present a composite awareness indices based on these conditions, highlighting the individual, household as well as community-based factors

## Methods

### Study setting

This study was conducted at the Agincourt Heath and Demographic Surveillance System (HDSS) site, located in the rural northeast of South Africa close to the border with Mozambique. The HDSS started in 1992 and currently covers an area of 450 km^2^ consisting of 31 villages with traditional and elected leadership.^17^ The HDSS includes a population of over 116 000 individuals from approximately 22 000 households. Within the HDSS area, there are two health centres and eight clinics, with three district hospitals about 25–60 km away. In contrast to other South African contexts, the healthcare facilities in and around the Agincourt HDSS provide general care and are not differentiated by disease or care types (eg, Maternal and Child Health, Infectious diseases). All clinics, health centres and district hospitals provide similar broad services including HIV testing and treatment, general NCD care as well as maternal and child healthcare. Consequently, residents of the area are not exposed to differential access to care type but do have differential access to care based on distance to the facilities. More details about the site can be found elsewhere in the literature.^17^

### Data sources

For our study, we use data from the baseline survey of the Health and Aging in Africa: A Longitudinal Study of an INDEPTH Community in South Africa (HAALSI) study which was a nested project based within the Agincourt HDSS population. HAALSI is an interdisciplinary study aiming to longitudinally monitor social, economic and biological risks for chronic health conditions, in a random sample of older adults in Agincourt, South Africa.^18^ The cohort which was established during the baseline data collection between November 2014 and November 2015 provides data on 5059 individuals aged 40 and over who permanently reside within the Agincourt surveillance area.^18^ Face-to-face interviews were conducted by experienced locally trained fieldworkers and supervisors using comprehensive household and individual computer-assisted personal interviews. Additionally, the study collected anthropometric measurements and biomarkers (biological samples) via point of care and dried blood spots, including venous blood by trained nurses during the individual visit at the household. The subsequent waves of data collection took place in 2018 and 2021. Detailed information concerning the sampling and data collection procedure for the HAALSI study is available in the HAALSI cohort profile.^18^

In this paper, we use self-reported data and objective biomarker measurements for hypertension, diabetes, dyslipidaemia and HIV to estimate awareness of a chronic condition. These data were collected in each wave by trained fieldworkers and/or nurses as part of the study. Our study focuses on awareness in the baseline wave of the study—even though additional waves are available—because our objective is to explore the determinants of awareness on a representative sample of the population. Awareness in the follow-up waves is likely to be influenced by participation in the study, and therefore, does not represent the normal trajectory of awareness. Additionally, the survey collected a large range of data including demographics, socioeconomic conditions, healthcare utilisation among others. We use the demographic and socioeconomic information—such as gender, age, marital status, living situation and economic standing—to explore the determinants of chronic condition awareness.

### Health conditions of interest and awareness

In this paper, we compare the self-reported and objective prevalence of hypertension, diabetes, dyslipidaemia and HIV during the baseline wave of the HAALSI study. As part of the study, HAALSI participants were asked whether a doctor, nurse or other health professional had ever diagnosed them or whether they were currently receiving treatment for the following conditions: high blood pressure, dyslipidaemia, stroke, heart failure, angina, myocardial infarction, diabetes, tuberculosis, HIV infection and kidney disease. Online supplemental table A1 presents the questions used in HAALSI for the conditions of interest.

In addition, the study measured high blood pressure and performed laboratory tests to measure high blood sugar, dyslipidaemia and HIV. This allowed us to compare self-reported with objective measurements to define awareness for a subset of chronic conditions. In particular, our study focuses on the awareness of hypertension (blood pressure ≥140/90 mm Hg), dyslipidaemia (total cholesterol >6.21 mmol/L or low-density lipoprotein (LDL)>4.1 mmol/L or high-density lipoprotein (HDL)<1.19 mmol/L or triglycerides >2.25 mmol/L), diabetes (elevated glucose—non-fasting ≥11.1 mmol/L or fasting ≥7 mmol/L) and HIV.

Using the self-reported and objective measures of the conditions above, we define an individual as being aware of their chronic condition if the positive self-reported status of a condition matched the status of the same condition on the objective measures. In this definition, individuals without the condition cannot be aware of the condition and are, therefore, discarded from our subsequent analysis on sociodeterminants of awareness. Using this information, we also include as an outcome whether individuals are aware of any of these conditions.

As a final outcome, we computed a simple chronic condition awareness index (CCAI) for each individual. There are many approaches to construct an index and we opted for the simplest approach of calculating the proportion of conditions that a person is aware of relative to the number of conditions they suffer. This approach follows that used by Saha *et al*^19^ which created an index for awareness of schizophrenia. However, it is important to note here that this area of the literature is limited since most studies explore awareness of specific conditions and there are no established measures of general awareness. The formula for the computation of the index is shown below and gives us the fraction of conditions that a person has which they are aware of.

$$\begin{array}{ll} CCAI= & Number\;of\;Conditions\;Aware\;of/\\ Number\;of\;Conditions\;participant\;has \end{array}$$

The advantages of constructing this index are its simplicity and intuitive interpretations. However, there are also limitations in that we assume that all conditions have equal weights, while it could be desirable to place greater weight on specific conditions. Second, this approach considers that all conditions may have the same relevance which is not necessarily the case. Future research should explore further how to create broader measures of awareness for chronic conditions that incorporate a set of chronic conditions.

### Statistical analysis

We begin our analysis by providing a general description on the different chronic conditions, their differences according to general demographic characteristics and the awareness of each condition. We then conduct multivariate logistic regressions where being aware of any or a specific condition is the outcome in a regression aiming to explore the socioeconomic determinants of awareness. We present five models: one for any condition and then one for HIV, hypertension, diabetes and dyslipidaemia. For this, we explore the association between awareness on the various chronic conditions and a set of individual, household and community factors. Finally, for the CCAI, we use a linear multivariate regression model. For the index, we use a linear model because it is a continuous measure from 0 to 1 reflecting the proportion of conditions a person is aware of relative to the number of conditions. Therefore, the most appropriate model is one that is linear in parameters, and not a logistic model since the outcome is not a dichotomous binary model.

To explore the determinants of awareness we identified individual, household and community-level factors. All of them are based on common factors identified by the literature as relevant in the relationship between health behaviours and health.^20^,^24^ In terms of individual factors, we have included age categorised into 10-year age groups, gender, years of education divided into three categories (no formal education, 1–7 years of education and more and 8 or more years of education), marital status (never married, separated or divorced, widowed and currently married) and their employment status distinguishing those who are not working, employed (part or full time) and retired.

With regard to household characteristics, we include the household composition (living alone, living with one person, living with two or more persons) and the socioeconomic status of the household defined as the monthly consumption per capita tertiles.^23 24^ A household is this context is defined as a group of individuals residing in a location who share a common meal. We further investigate the relationship of each household member with the participant in a second model. Finally, in terms of community factors, we include the distance to the closest healthcare facility in three categories (less than 1.5 km, between 1.5 and 3 km, more than 3 km). These categories were chosen as the distance to nearest clinic for 66% of the study population is between 1 and 3 km (online supplemental figure A1).^25^

For all analysis, we estimate the robust SEs clustered at the household level since outcomes are likely to be correlated between members of a household. All statistical analyses were performed by using Stata Statistical Software (STATA) V.14.2 (Stata).

## Results

### Prevalence of chronic conditions

**Figure 1:**
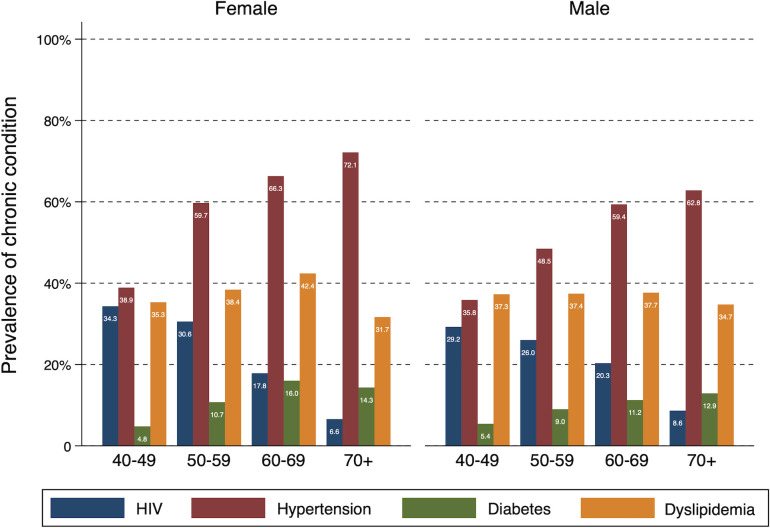
Prevalence by gender and age group for chronic conditions.

The HAALSI baseline survey dataset collected information on a total of 5059 participants 40 years old and older. A detailed description of the demographic profile of the HAALSI baseline sample is presented elsewhere.^18^ At baseline, 4052 individuals (80%) suffered from at least one of the following conditions: HIV, hypertension, diabetes and dyslipidaemia. Figure 1 shows the age-specific and gender-specific prevalence of each of the chronic conditions. In the case of HIV, we found that 21% of women and 20% of men were HIV positive. As the figure shows, the prevalence of HIV decreases with age. Furthermore, we found that 62% of women and 55% of men suffered from hypertension and the figure shows an increasing prevalence with age. In the case of diabetes, approximately 13% of women and 11% of men had diabetes at baseline and the prevalence increases over time but we can see a small decrease in the females aged 70 and over. Finally, for dyslipidaemia, we found that 43% of women, and 45% of men had dyslipidaemia. We observe an increasing trend for both men and women with age, with a slight drop for those aged 70+. Online supplemental table A2 shows more detailed results on the prevalence of chronic conditions and the differences across the set of individual, household and community factors that we explore.

### Awareness of chronic conditions

Table 1 presents the fraction of individuals with any or specific condition that are aware of their chronic conditions. Generally, 77.9% of the total sample are aware of some of the conditions they have. Specifically, 83.4% are aware of their HIV-positive status; 85.4% are aware of their hypertensive status, 75.3% are aware they have diabetes, whereas just 10.2% are aware of having dyslipidaemia.

**Table 1:** General prevalence and awareness

	Any	HIV	Hypertension	Diabetes	Dyslipidaemia
Number of prevalent individuals	4052	1055	2901	559	1862
% of awareness	77.9	83.4	85.4	75.3	10.2

**Figure 2:**
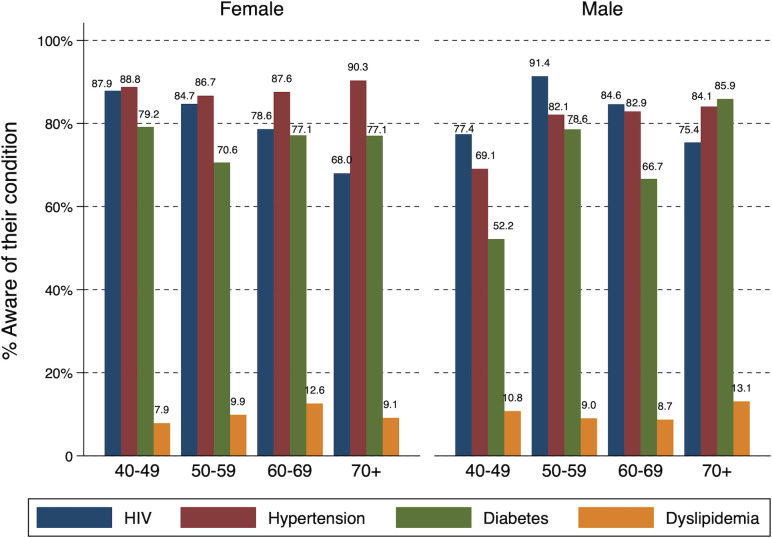
Age-specific and gender-specific awareness by condition.

Figure 2 presents detailed information on age-specific and gender-specific awareness of the different chronic conditions. In line with the overall statistics above, there is generally a high awareness of the different conditions. However, we found heterogeneity in the levels across age and gender. From the figure, we observe that males in their 40s are generally less aware of their conditions than other population subgroups. In case of diabetes, their awareness is approximately at 50%. The figure also highlights how awareness of HIV decreases for females as they get older. In the case of hypertension, we see little differences in awareness rates across gender and different age groups. There is, however, a slight increase with age among males, with significant differences between the 40–49 age subgroup and the 50+ age group. Finally, for dyslipidaemia, awareness generally increases for females as they age, but drops again with the 70+ subgroup. However, the reverse happens for males: awareness decreases with age, and increases with the 70+ subgroup. Online supplemental table A3 presents the detailed awareness statistics across all individual, household and community factors included in the analysis.

### Sociodemographic determinants of awareness

Table 2 presents the results (estimated adjusted ORs (aOR) with 95% CIs in parenthesis) for the multivariate logistic regression showing the factors associated with being aware of the conditions of interest . The table shows all five multivariate logistic regressions. The model for associations with any health condition shows that gender, age, household living composition, consumption per capita, as well as distance to nearest health facility are associated with being aware of any of the conditions discussed. Specifically, males have lower odds (aOR 0.66, 95% CI 0.56 to 0.78) of being aware of any of their chronic conditions. We observe that being aware of any of the conditions increases with age, with the 70+ having the highest OR (aOR 1.66, 95% CI 1.26 to 2.19), whereas individuals living with one more persons have higher odds of being aware of their chronic condition than individuals living alone (aOR 1.69, 95% CI 1.18 to 2.40). We explore the household composition further in online supplemental table A4 and show that individuals living with a child in their household are those who are particularly more aware thus suggesting that there may be a channel through the children that can increase awareness of diseases. This can be either through information or also through practical mechanisms like helping parents access healthcare. Additionally, we observe a positive gradient with increase in consumption per capita tertiles and awareness, that is, persons from the richest households (T3) have higher odds of being aware of any condition (aOR 1.81, 95% CI 1.46 to 2.26) compared to those in T2 (aOR 1.51, 95% CI 1.24 to 1.82) and T1 (the reference group). The results further show that persons who are within more than 3 km of a health facility are less likely (aOR 0.79, 95% CI 0.67 to 0.94) of being aware of their chronic condition compared with those who are less than 3 km away (p<0.01).

**Table 2:** Determinants of chronic condition awareness

	Any condition	HIV	Hypertension	Diabetes	Dyslipidaemia
	OR (95% CI)	OR (95% CI)	OR (95% CI)	OR (95% CI)	OR (95% CI)
Gender					
Female	1	1	1	1	1
Male	0.66 (0.56 to 0.78)*	1.10 (0.75 to 1.62)	0.54 (0.42 to 0.68)*	0.85 (0.52 to 1.39)	0.90 (0.63 to 1.27)
Age group					
40–49	1	1	1	1	1
50–59	1.51 (1.20 to 1.90)*	1.80 (1.12 to 2.88)†	1.41 (1.00 to 2.00)	1.15 (0.52 to 2.51)	1.17 (0.71 to 1.92)
60–69	1.44 (1.11 to 1.86)‡	1.17 (0.68 to 2.02)	1.53 (1.04 to 2.25)†	1.02 (0.46 to 2.28)	1.65 (0.96 to 2.84)
70+	1.66 (1.26 to 2.19)*	0.83 (0.44 to 1.59)	2.04 (1.36 to 3.08)*	1.81 (0.75 to 4.33)	2.04 (1.14 to 3.66)†
Years of education					
No education	1	1	1	1	1
Some primary (1–7 years)	1.18 (0.99 to 1.41)	1.56 (1.02 to 2.36)†	1.44 (1.11 to 1.88)‡	1.02 (0.63 to 1.66)	1.22 (0.84 to 1.78)
Secondary or more (8+years)	1.16 (0.91 to 1.46)	2.01 (1.14 to 3.53)†	1.26 (0.89 to 1.78)	0.93 (0.45 to 1.94)	0.95 (0.57 to 1.60)
Employment status					
Not working	1	1	1	1	1
Employed (part or full time)	1.02 (0.82 to 1.28)	1.16 (0.72 to 1.87)	1.25 (0.89 to 1.75)	1.57 (0.78 to 3.19)	1.02 (0.65 to 1.58)
Retired	1.16 (0.97 to 1.40)	1.50 (0.96 to 2.35)	1.20 (0.93 to 1.54)	2.87 (1.68 to 4.93)*	0.44 (0.29 to 0.68)*
Marital status					
Never married	1	1	1	1	1
Separated/divorced	1.45 (0.99 to 2.12)	2.24 (1.07 to 4.69)†	0.91 (0.52 to 1.60)	1.46 (0.45 to 4.74)	0.89 (0.37 to 2.13)
Widowed	1.33 (0.92 to 1.92)	1.24 (0.63 to 2.46)	1.19 (0.69 to 2.07)	1.68 (0.56 to 5.04)	0.85 (0.37 to 1.93)
Currently married	1.15 (0.81 to 1.62)	1.32 (0.68 to 2.59)	1.26 (0.75 to 2.12)	1.48 (0.53 to 4.09)	0.91 (0.42 to 1.96)
HH living composition					
Living alone	1	1	1	1	1
Living with 1 other person	1.69 (1.18 to 2.40)‡	1.08 (0.54 to 2.16)	2.01 (1.22 to 3.31)‡	1.16 (0.44 to 3.06)	1.33 (0.65 to 2.73)
Living in 3–6 person household	1.48 (1.11 to 1.96)‡	1.74 (0.97 to 3.13)	1.35 (0.92 to 1.97)	1.19 (0.51 to 2.82)	1.59 (0.86 to 2.95)
Living in 7+ person household	1.54 (1.12 to 2.12)‡	1.31 (0.68 to 2.51)	1.78 (1.16 to 2.74)‡	1.37 (0.53 to 3.54)	2.23 (1.14 to 4.38)†
Consumption per capita tertiles					
T1 (low)	1	1	1	1	1
T2 (medium)	1.51 (1.24 to 1.82)*	1.23 (0.81 to 1.88)	1.35 (1.03 to 1.78)†	1.53 (0.89 to 2.61)	2.88 (1.74 to 4.77)*
T3 (high)	1.81 (1.46 to 2.26)*	1.49 (0.90 to 2.47)	1.46 (1.08 to 1.97)†	2.25 (1.22 to 4.17)‡	6.10 (3.66 to 10.15)*
Distance to nearest clinic					
Less than 1.5 km	1	1	1	1	1
Between 1.5 and 3 km	1.29 (1.05 to 1.57)†	1.23 (0.80 to 1.89)	1.21 (0.91 to 1.61)	1.16 (0.67 to 1.99)	1.63 (1.12 to 2.37)†
More than 3 km	0.79 (0.67 to 0.94)‡	0.97 (0.65 to 1.43)	0.73 (0.57 to 0.92)‡	0.80 (0.50 to 1.30)	0.84 (0.57 to 1.24)

Note: Table presents odds ratiosORs with 95% confidence intervalCIs estimated with household robust clustered standard errorSEs in parenthesis.

* p<0.001.

† p<0.05

‡ p<0.01

In the model for HIV, table 2 shows that, years of education as well as marital status are associated with being aware of being HIV positive. Interestingly, the results further show that men have higher odds (aOR 1.10, 95% CI 0.75 to 1.62), of being aware than women though not statistically significant (p=0.6186). Those aged 40–69 have higher odds and more likely to be aware of their HIV-positive status than those aged 70+. Persons with more years of education have significantly higher odds (aOR 2.01, 95% CI 1.14 to 3.53) of being aware than persons with less years of education (p<0.05). We observe a positive odds gradient as number of years of education increases (p<0.05). More so, individuals who never married have lower odds of being aware than those who are currently married, separated/divorced or widowed. We observed that, separated or divorced persons have significantly higher odds (aOR 2.24, 95% CI 1.07 to 4.69) than all others in the group (p<0.05). Additionally, we observe a positive odds gradient of being aware of one’s HIV-positive status with increasing consumption per capita, and a negative gradient with increasing distance to nearest health facility, though these were not statistically significant.

Adjusted logistic analysis for hypertension showed that gender, age, education, household living composition, as well as consumption per capita and distance to nearest health facility are factors associated with being aware of one’s hypertensive status. Men are less likely (aOR 0.54, 95% CI 0.42 to 0.68) to be aware than women and being aware increases with age. More so, those who have no education are less likely to be aware compared with those who have some primary or more education (aOR 1.44, 95% CI 1.11 to 1.88). Additionally, persons living with one or more household members have higher odds of being aware, than those living alone—again mainly motivated by children in the household. Persons from highest ranked consumption per capita households (T3) have higher odds (aOR 1.46, 95% CI 1.08 to 1.97), than persons from the lower ranked households (T1) (p<0.05). Also, individuals living farthest (more than 3 km) from a health facility have lower odds of being aware (aOR 0.73, 95% CI 0.57 to 0.92), than those closer (p<0.01).

In the model on diabetes, we found that employment status and consumption per capita were factors associated with being aware of diabetes chronic condition. Retired persons are more likely to be aware of their diabetic condition (aOR 2.87, 95% CI 1.68 to 4.93) compared with the currently employed subgroup, and the currently employed are also more likely to be aware of their diabetic condition compared with the non-working subgroup although this association is not significant. More so, we observe a positive gradient with consumption per capita. Persons in the higher ranked tertiles (T3) of the consumption per capita have higher odds and more likely to be aware of their diabetic condition (p<0.01) compared with those in the lower ranked tertiles (T1) subgroups. Additionally, we observe men are less likely to be aware compared with women; those living alone are also less likely to be aware compared with those living with one or more persons, and those who live farthest from a health facility are less likely to be aware of their diabetic condition, though these are not statistically significant.

In the multivariate model on dyslipidaemia, we found older age, employment, household living composition, high consumption per capita, as well as medium distance to nearest health facility as factors associated with being aware of dyslipidaemia. Awareness increases with age, and those who are retired are less likely to be aware compared with those working or not working. Also, we observe increasing awareness with increasing number of household member composition, similarly stronger for older household members living with a child. More so, persons from higher ranked consumption per capita household tertiles have higher odds (aOR 6.10, 95% CI 3.66 to 10.15) of being aware than those from lower ranked households (p<0.001), and persons who live farthest from a health facility are less likely to be aware compared with those who live closer.

### Awareness indices

**Figure 3:**
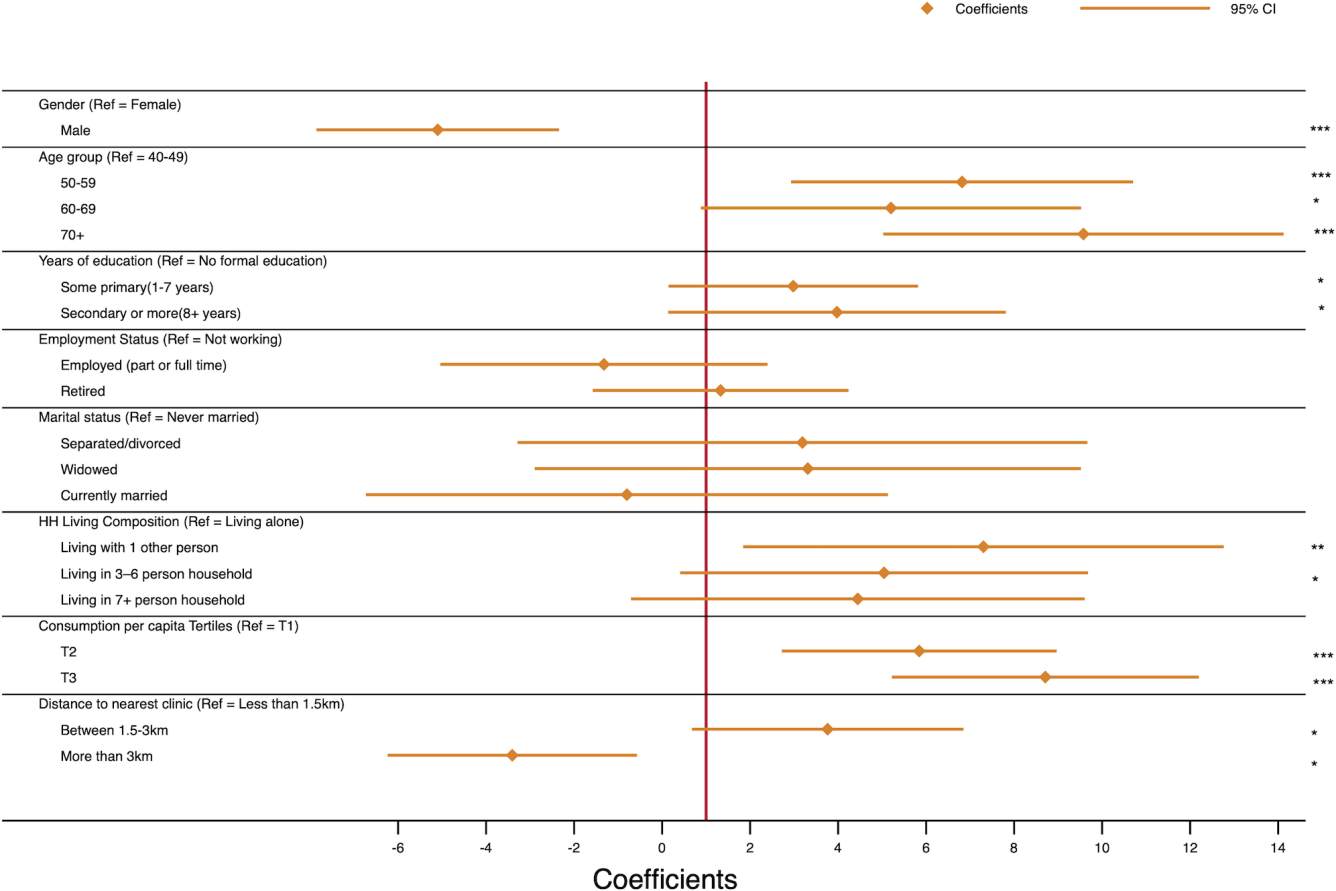
Factors associated with awareness index. *p<0.05, **p<0.01, ***p<0.001.

Using our constructed awareness index, we found the mean index to be 0.64 (95% CI 0.63 to 0.65), indicating that on average people were aware of 64% of the conditions they suffered from. Online supplemental table A5 presents detailed means with 95% CIs for the various individual, household and community covariates discussed in this paper. A multivariate linear regression was conducted to determine related factors associated with the indices computed. We found gender, age, education, household living composition, consumption per capita, as well as distance to nearest health facility factors associated with the awareness index. Figure 3 shows the detailed plot of the various factors, and their corresponding regression coefficients.

## Discussion

We find that despite the generally high awareness of chronic conditions (81% for women and 74% for men) in the cohort, there are important differences by age, gender and socioeconomic status. Our findings show that targeted approaches may be required to increase awareness of chronic conditions, and therefore, address the NCD epidemic across the globe.

The findings of the paper show that 83% of women and 84% of men are aware of their HIV-positive status, whereas 88% of women and 81% of men are aware of their hypertensive status. Awareness of diabetes was, however, comparatively low (76% for women and 75% for men). These rates are higher than previously published work in the region.^26 27^ The increased awareness could possibly be due to previous studies in the region which focused on hypertension and stroke.^2628^,^30^ Women are more aware of their condition than men in line with findings from Payne *et al*.^31^,^33^ Available evidence suggests a higher health service utilisation for women for reasons such as care giving, which generally increases their knowledge of those conditions and eventual awareness. Also, it is suggested that men use health services less due to their perceived strength and courage.^34^ Our findings further suggest that generally, increased level of education is highly associated with increased awareness for HIV, hypertension and diabetes.^18^ Men and women with some primary or more education are generally more aware than those with no formal education, suggesting the need to adopt health education strategies that can resonate with those with no formal education as well. We also observe a very low awareness of dyslipidaemia (10% for both men and women), possibly due to lack of explicit care and screening of the condition, especially in the rural population under surveillance. Unlike dyslipidaemia, blood pressure and blood glucose measurement are standard screening services provided to patients who visit the health facility.

Though a high proportion of old adults are aware of their HIV status, there are still some sociodemographic differentials accounting for over 15% not being aware despite the many efforts over the years to create awareness about HIV, especially in sub-Saharan Africa. We found that adults older than 60 years are less likely to be aware of their HIV-positive status compared with those under 60 years of age. This could be due to lower risk perception, and the belief that HIV is only prevalent among the younger population. Though South Africa is nationally on track^35^ in the achievement of the first 90, and 95 of the 90-90-90, and 95-95-95 targets, respectively, of the Joint United Nations Programme on HIV/AIDS (UNAIDS),^36^ our results have shown that subpopulation groups such as discussed above will require more efforts in reaching the UNAIDS goal of being aware of HIV-positive status. Strategic and targeted efforts are, therefore, required to reach more adults, particularly the 60+; and the uneducated, to increase HIV awareness and eventually promote self-management.

We found factors such as gender, age, education, household living composition, consumption per capita, as well as distance to nearest health facility significantly associated with being aware of hypertensive status. Age group is positively associated with being aware of hypertension, while some form of education is positively associated with being aware of hypertensive condition. Due to the natural biological degeneration associated with ageing, the older population may eventually be compelled to attend a health facility, especially those living with other family members. Available family support systems could help improve health literacy and result in awareness. Younger men, the uneducated, as well as persons from the lowest ranked consumptions per capita households, are a likely group that needs a more rigorous approach to increase knowledge and awareness in general with regard to hypertension awareness.

Age is generally recognised as one of the most important risk factors for diabetes.^37 38^ We observe a higher prevalence among the 70+. The results further show a higher proportion of those retired as well as those from rich households being aware of their diabetic status. This could possibly be due to the fact that retired individuals often have more time, allowing them to prioritise healthcare visits and screenings, while those from rich households are able to afford transportation to a health facility for screening, leading to higher awareness. Diabetes education needs to be intensified, with strategic educational intervention campaigns to improve knowledge and increase awareness. Diabetes awareness is the second lowest among the four conditions analysed. The International Diabetes Federation (2021) reports that 81% of adults with diabetes live in low-income and middle-income countries, and that diabetes accounted 6.7 million deaths in 2021 worldwide. It is, thus, imperative that targeted pragmatic steps are required to drive knowledge and eventually increase awareness in rural sub-Saharan Africa, especially targeting the 40–49 age group, the unemployed, as well as the poorest households (lowest ranked consumption per capita). These efforts must be targeted at creating awareness which will eventually promote self-management among the older adults living with diabetes in rural South Africa.

The prevalence of dyslipidaemia in the cohort is about 43%, but the observed significantly low awareness of dyslipidaemia in line with the findings of Reiger *et al*^16^ in the cohort is of great concern. Dyslipidaemia is a risk factor for several cardiovascular diseases (CVDs),^39^,^41^ and therefore, needs pragmatic efforts to create awareness at the various health facilities, and possibly included in frequently screened conditions. It is, therefore, imperative to provision resources, and policies to promote and increase awareness of the condition which will eventually reduce the CVD burden on the already constrained health systems. Knowing and being aware of the complications of dyslipidaemia could, further, lead to proper self-management, early care seeking and improve the level of awareness of associated CVDs that could result from the condition. Finally, across several conditions, we found that living with a child leads to higher likelihoods of awareness. This can be due to information being transmitted from child to parent, or through practical considerations like children helping their parents access healthcare services.

We find similar findings based on the general awareness index. Men, as well as persons who live more than 3 km aware from a health facility, show negative association. The index is positively associated and generally increases with age, education and household consumption per capita. The over 70-year olds tend to have 9.6 times higher index (p>0.001) compared with the 40–49 age group. The index for those with some form of education (primary or more) is estimated to be over three times more than that of those with no education. Also, those living with at least one additional person are expected to have higher index compared with those living alone. Whereas, those from higher ranked consumption per capita households have higher index compared with those from lower ranked consumption per capita households. The results from the awareness indices further reinforce the need to implement targeted strategies for different subpopulation groups to promote awareness, and consequently improve self-management of chronic conditions.

Though, we find high level of awareness for HIV, hypertension and diabetes, the potential cost of not being aware cannot be overemphasised. Lack of awareness could lead to delayed diagnosis and initiation of treatment, which may result in the progression of the chronic condition, increased complications and mortality risk.^42 43^ Additionally, late-stage interventions could be more resource-intensive compared with early preventive measures. Unmanaged chronic conditions as a result of not being aware may also lead to increased likelihood of emergency room visits and hospitalisations, further burdening the limited healthcare resources, especially in such resource-poor communities.^44^,^46^

There are, however, some limitations to consider about this study. One main limitation of our study is that the behavioural patterns for healthcare seeking of infectious diseases like HIV are likely to be different to those from lifestyle diseases such as diabetes. This can influence the level and the gradients of awareness since participants may tend to travel outside of their community for diseases that are affected by stigma. In our analysis, we adjust for distance to the nearest facility, but this may not be necessarily the facility that participants attend. However, the concern raised from this limitation is reduced when considering the overall high awareness of HIV which could also be influenced by government policies and campaigns that are unrelated to the healthcare facilities used by participants.

A second limitation of this study is the construction of the awareness index. Currently, there are no approaches in the literature that have been established to evaluate general awareness of chronic conditions since most papers focus on the awareness of specific conditions. For this paper, we relied on the simplest method to construct the index as the proportion of conditions a person is aware of. While this allows for intuitive interpretations, there are some challenges and limitations of its construction. First, our index assumes that all conditions are similar while this may not necessarily be the case from the individual or policy makers perspective. Second, the index assumes that the proportion of awareness is relevant while it is possible to argue that the composition of awareness could be relevant for improving health behaviours. Finally, the index focuses on the proportion while what may be relevant is the absolute number of conditions one is aware of. All these challenges, however, should motivate future research to explore and analyse different approaches in computing general indices of awareness.

A third limitation is that for this study we limit our analysis to the baseline despite HAALSI being a longitudinal study. The dynamics of chronic condition awareness are relevant, and the current literature suggests that awareness can lead to appropriate management of chronic conditions and potentially reduced mortality.^47 48^ This should be undertaken in future research. One limitation of HAALSI for that is that awareness can be influenced by participation in the survey, therefore, the trajectories of awareness may not represent what occurs to a representative sample.

## Conclusion

This study has shown that being aware of one’s chronic condition is multifaceted and influenced by different sociodemographic characteristics. The younger among the older population, with more education years, who have been or are in a marital relationship, were found to be more aware of their HIV-positive status, possibly due to lower risk perception by the older population, as well as the fact that most awareness and educational campaigns tend to focus more on the younger population.^49^,^51^ The younger population seems to have a higher perception of risk than the older population. Nyirenda *et al*^50^ found that only 9.4% of adults aged 50 years and over had a ‘high-risk’ HIV perception in a study comprising both rural and urban participants in South Africa. Additionally, contrary to general belief, we found a slightly higher proportion of men than women being aware of their HIV-positive status indicating a possible shift in the gender dynamics as far as HIV is concerned, and thus suggests the need for targeted messages to reach the older female population in particular. Awareness of diabetes was found to be very low and would require extra targeted efforts to ensure general education and creation of awareness especially among the younger elderly. Similarly, the findings from this study suggest the need to intensify efforts to reach the younger among the older population on hypertension awareness and promote healthy lifestyle. More so, given the very low awareness of dyslipidaemia, it is critical for policy implementation, and establishment of practical steps to promote awareness of the condition, which could further enhance awareness of other CVDs. It is, thus, urgent to evaluate the effect of health education in rural South Africa, find its related factors, and develop effective health education strategy in order to control chronic diseases among various target subgroups.

## supplementary material

10.1136/bmjph-2023-000315 online supplemental file 1

## Data Availability

Data are available in a public, open access repository. Data are available on reasonable request.
