# Supplementary material for: Sociodemographic disparities in awareness of chronic conditions: an observational study among older persons in rural north-east of South Africa
Source: BMJ Public Health. 2024 Mar 13;2(1):e000315. doi: 10.1136/bmjph-2023-000315 (PMC11812760; doi:10.1136/bmjph-2023-000315)
Supplement: online supplemental file 1 [file bmjph-2-1-s001.pdf]

Supplementary Materials - Appendix

Table A1. Questions used to evaluate self-reported chronic conditions

Condition	Questions
Hypertension[1]	CM001 Has a doctor, nurse, or other healthcare worker ever measured your blood pressure<br><br>CM002 Have you ever been told by a doctor, nurse, or other healthcare worker that you have high blood pressure or hypertension?
Diabetes[2]	CM007 Has a doctor, nurse, or other healthcare worker ever measured your urine or blood for diabetes?<br><br>CM012 Are you currently taking any herbal or traditional remedy for your diabetes?
HIV[3]	CM013 Have you ever been tested for HIV?<br><br>CM093 Do you know your status?<br><br>CM014 Have you ever tested positive for HIV?<br><br>CM018 Are you currently taking any herbal or traditional remedy for HIV?<br><br>CM019 Have you ever been counselled by a doctor, nurse, ART/HIV counsellor, or other health worker on how you can avoid passing on HIV to others?
Dyslipidemia[4]	CM045 Has a doctor, nurse, or other healthcare worker ever measured your cholesterol?<br><br>CM046 Have you ever been told by a doctor, nurse, or other healthcare worker that you have high cholesterol<br><br>CM049 Are you currently taking any herbal or traditional remedy for high cholesterol?

[1] A person is categorized as self-reporting aware hypertension if they responded YES to any of these questions.

[2] A person is categorized as self-reporting aware diabetes if they respond YES to any of the questions listed.

[3] A person is categorized self-reporting aware HIV if they respond YES to any of the related questions provided.

[4] A person is categorized as self-reporting aware dyslipidemia if they respond YES to either question.

Table A2. Prevalence of chronic conditions in HAALSI sample

	ANY			HIV			Hypertension			Diabetes			Dyslipidemia		
	n (%)	*N*		*n (%)*	*N*		*n (%)*	*N*		*n (%)*	*N*		*n (%)*	*N*	
**Gender**															
Female	483 (17.8)	2230		582 (21.5)	2713		1653 (61.9)	2672		323 (12.9)	2509		1001 (42.9)	2331	
Male	524 (22.3)	1822	***	473 (20.2)	2346	***	1248 (54.7)	2281	***	236 (11.0)	2143		861 (44.8)	1920	
**Age Group**															
40-49	254 (27.4)	674		297 (32.0)	928		348 (38.6)	902		47 (5.6)	833		336 (44.8)	750	
50-59	263 (18.6)	1152	***	404 (28.6)	1415	***	775 (56.0)	1385	***	141 (10.9)	1289	***	537 (45.5)	1179	***
60-69	214 (16.5)	1082		247 (19.1)	1296		815 (63.8)	1278		177 (14.6)	1216		519 (46.4)	1119	
70+	276 (19.4)	1144		107 (7.5)	1420		963 (69.4)	1388		194 (14.8)	1314		470 (39.1)	1203	
**Years of Education**															
No formal education	457 (19.7)	1857		436 (18.8)	2314		1404 (62.0)	2264		243 (11.4)	2134		828 (42.2)	1960	
Some primary (1-7 years)	318 (18.4)	1406	*	373 (21.6)	1724	***	981 (57.8)	1697	***	218 (13.5)	1617		669 (44.9)	1489	
Secondary or more (8+ years)	232 (22.7)	789		246 (24.1)	1021		516 (52.0)	992		98 (10.9)	901		365 (45.5)	802	
**Employment**															
Not working	554 (20.2)	2186		615 (22.4)	2740		1547 (57.9)	2674		317 (12.7)	2494		995 (43.8)	2273	
Employed (part or full time)	177 (21.8)	634		203 (25.0)	811	***	390 (49.4)	789	***	69 (9.4)	734		333 (50.5)	659	***
Retired	276 (18.3)	1232		237 (15.7)	1508		964 (64.7)	1490		173 (12.1)	1424		534 (40.5)	1319	
**Marital status**															
Never married	93 (31.6)	201		77 (26.2)	294		110 (39.0)	282		22 (8.7)	252		85 (39.0)	218	
Separated / divorced	121 (18.6)	529	***	207 (31.8)	650	***	353 (55.2)	640	***	59 (9.9)	594		232 (42.3)	548	***
Widowed	267 (17.3)	1273		350 (22.7)	1540		961 (64.0)	1502		185 (13.0)	1421		514 (39.6)	1299	
Currently married	526 (20.4)	2049		421 (16.3)	2575		1477 (58.4)	2529		293 (12.3)	2385		1031 (47.2)	2186	
**Household composition**															
Living alone	103 (19.3)	431		153 (28.7)	534		293 (56.7)	517		52 (10.8)	483		171 (38.9)	440	
Living with 1 other person	116 (21.6)	422		108 (20.1)	538		310 (59.6)	520		68 (14.0)	486		176 (39.3)	448	***
Living in 3-6 person household	505 (20.7)	1933		500 (20.5)	2438		1397 (58.2)	2399		266 (11.9)	2242		882 (43.4)	2031	
Living in 7+ person household	283 (18.3)	1266		294 (19.0)	1549		901 (59.4)	1517		173 (12.0)	1441		633 (47.5)	1332	
**Consumption per capita tertiles**															
T1	341 (20.2)	1345		366 (21.7)	1686		908 (55.1)	1647		154 (9.9)	1561		616 (42.8)	1439	
T2	338 (20.0)	1348		353 (20.9)	1686	**	992 (59.8)	1660	**	178 (11.5)	1551	***	612 (43.1)	1421	
T3	328 (19.4)	1359		336 (19.9)	1687		1001 (60.8)	1646		227 (14.7)	1540		634 (45.6)	1391	
**Distance to nearest clinic**															
Less than 1.5km	410 (20.0)	1637		415 (20.3)	2047		1196 (59.4)	2013		222 (11.8)	1875		743 (43.5)	1707	
Between 1.5-3km	261 (20.2)	1033		289 (22.3)	1294		730 (57.7)	1266		144 (12.1)	1194		468 (42.8)	1094	
More than 3km	335 (19.5)	1380		350 (20.4)	1715		973 (58.2)	1671		193 (12.2)	1580		650 (44.9)	1447	

* p<.001, ** p<.01, *** p<.05

Table A3. Awareness of chronic conditions

	Any & Aware			HIV+ & Aware			Hypertensive & Aware			Diabetics & Aware			Dyslipidemia & Aware		
	n (%)	*N*		*n (%)*	*N*		*n (%)*	*N*		*n (%)*	*N*		*n (%)*	*N*	
**Gender**															
Female	1815 (81.4)	2230		483 (83.0)	582		1461 (88.4)	1653		244 (75.5)	323		101 (10.1)	1001	
Male	1342 (73.7)	1822	***	397 (83.9)	473		1016 (81.4)	1248	***	177 (75.0)	236		89 (10.3)	861	
**Age Group**															
40-49	486 (72.1)	674		248 (83.5)	297		279 (80.2)	348		31 (66.0)	47		31 (9.2)	336	
50-59	905 (78.6)	1152	***	353 (87.4)	404	**	658 (84.9)	775	**	104 (73.8)	141		51 (9.5)	537	
60-69	846 (78.2)	1082		202 (81.8)	247		696 (85.4)	815		129 (72.9)	177		56 (10.8)	519	
70+	920 (80.4)	1144		77 (72.0)	107		844 (87.6)	963		157 (80.9)	194		52 (11.1)	470	
**Years of Education**															
No formal education	1439 (77.5)	1857		341 (78.2)	436		1190 (84.8)	1404		181 (74.5)	243		74 (8.9)	828	
Some primary (1-7 years)	1110 (78.9)	1406		322 (86.3)	373	***	860 (87.7)	981	*	163 (74.8)	218		75 (11.2)	669	
Secondary or more (8+ years)	608 (77.1)	789		217 (88.2)	246		427 (82.8)	516		77 (78.6)	98		41 (11.2)	365	
**Employment**															
Not working	1685 (77.1)	2186		503 (81.8)	615		1307 (84.5)	1547		219 (69.1)	317		115 (11.6)	995	
Employed (part or full time)	483 (76.2)	634	*	175 (86.2)	203		330 (84.6)	390		52 (75.4)	69	***	41 (12.3)	333	**
Retired	989 (80.3)	1232		202 (85.2)	237		840 (87.1)	964		150 (86.7)	173		34 (6.4)	534	
**Marital status**															
Never married	143 (71.1)	201		62 (80.5)	77		87 (79.1)	110		14 (63.6)	22		9 (10.6)	85	
Separated / divorced	421 (79.6)	529	***	184 (88.9)	207		286 (81.0)	353	**	44 (74.6)	59		21 (9.1)	232	
Widowed	1041 (81.8)	1273		284 (81.1)	350		847 (88.1)	961		145 (78.4)	185		50 (9.7)	514	
Currently married	1552 (75.7)	2049		350 (83.1)	421		1257 (85.1)	1477		218 (74.4)	293		110 (10.7)	1031	
**Household composition**															
Living alone	324 (75.2)	431		125 (81.7)	153		233 (79.5)	293		40 (76.9)	52		17 (9.9)	171	
Living with 1 other person	351 (83.2)	422	**	89 (82.4)	108		280 (90.3)	310	**	54 (79.4)	68		22 (12.5)	176	
Living in 3-6 person household	1520 (78.6)	1933		430 (86.0)	500		1185 (84.8)	1397		199 (74.8)	266		93 (10.5)	882	
Living in 7+ person household	962 (76.0)	1266		236 (80.3)	294		779 (86.5)	901		128 (74.0)	173		58 (9.2)	633	
**Consumption per capita tertiles**															
T1	979 (72.8)	1345		295 (80.6)	366		756 (83.3)	908		101 (65.6)	154		25 (4.1)	616	
T2	1073 (79.6)	1348	***	296 (83.9)	353		857 (86.4)	992		134 (75.3)	178	***	59 (9.6)	612	***
T3	1105 (81.3)	1359		289 (86.0)	336		864 (86.3)	1001		186 (81.9)	227		106 (16.7)	634	
**Distance to nearest clinic**															
Less than 1.5km	1280 (78.2)	1637		343 (82.7)	415		1028 (86.0)	1196		170 (76.6)	222		67 (9.0)	743	
Between 1.5-3km	845 (81.8)	1033	***	246 (85.1)	289		641 (87.8)	730	*	112 (77.8)	144		67 (14.3)	468	**
More than 3km	1031 (74.7)	1380		291 (83.1)	350		807 (82.9)	973		139 (72.0)	193		56 (8.6)	650	

*** p<.001 ** p<.01 * p<.05

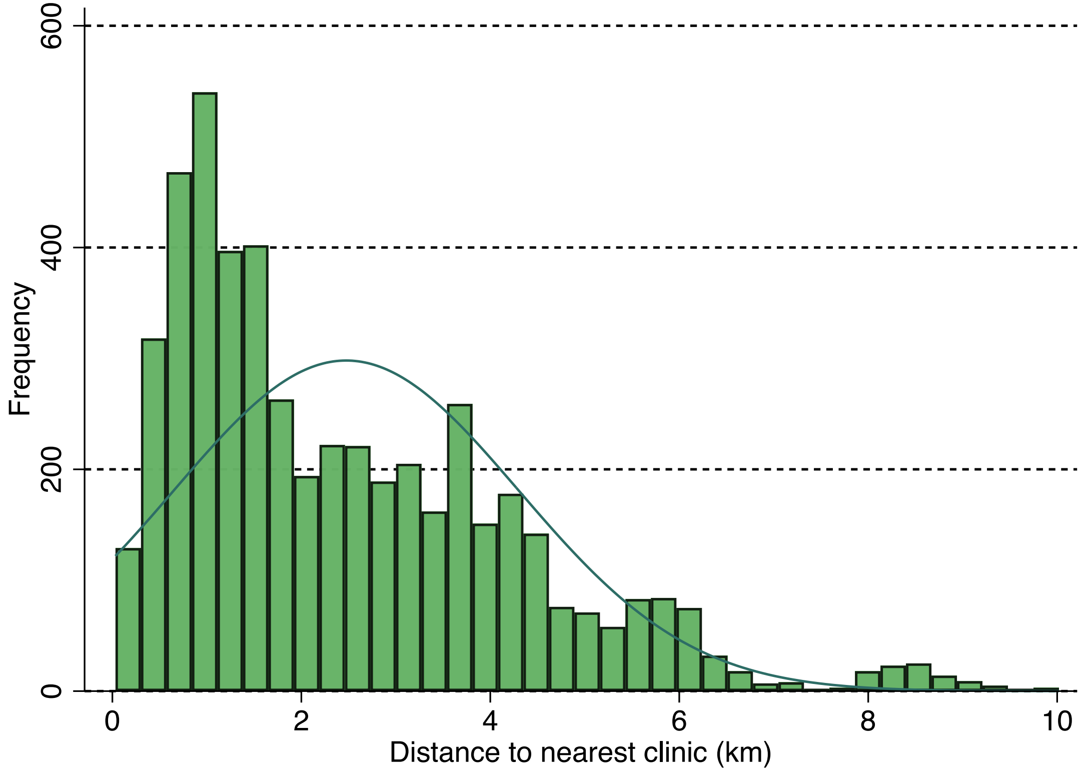

Figure A1. Distribution of distance to nearest health facility for study participant

Table A4. Determinants of chronic condition awareness with Household relations

	Any Condition	HIV	Hypertension	Diabetes	Dyslipidemia
Variables	Odds ratios (95% CI)	Odds ratios (95% CI)	Odds ratios (95% CI)	Odds ratios (95% CI)	Odds ratios (95% CI)
**Gender**					
Female	1	1	1	1	1
Male	0.61(0.51-0.74) ***	1.03(0.69-1.55)	0.54(0.41-0.70) ***	0.85(0.50-1.44)	0.74(0.50-1.11)
**Age group**					
40-49	1	1	1	1	1
50-59	1.50(1.19-1.90) ***	1.72(1.07-2.75) *	1.38(0.97-1.95)	1.41(0.65-3.07)	1.21(0.73-1.98)
60-69	1.43(1.10-1.86) **	1.09(0.64-1.88)	1.45(0.98-2.13)	1.11(0.50-2.47)	1.72(1.00-2.97)
70+	1.68(1.27-2.22) ***	0.78(0.41-1.49)	1.82(1.21-2.74) **	1.60(0.68-3.79)	2.05(1.14-3.69) *
**Years of education**					
No education	1	1	1	1	1
Some primary(1-7 years)	1.16(0.97-1.39)	1.59(1.07-2.38) *	1.43(1.11-1.84) **	1.16(0.73-1.84)	1.20(0.83-1.73)
Secondary or more(8+ years)	1.13(0.89-1.44)	1.86(1.08-3.18) *	1.10(0.80-1.53)	1.19(0.59-2.39)	0.97(0.59-1.59)
**Employment Status**					
Not working	1	1	1	1	1
Employed (part or full time)	1.01(0.81-1.27)	1.14(0.71-1.84)	1.19(0.85-1.66)	1.29(0.65-2.54)	0.99(0.64-1.54)
Retired	1.15(0.95-1.38)	1.55(0.99-2.43)	1.16(0.90-1.49)	3.09(1.82-5.27) ***	0.41(0.27-0.63) ***
**Marital status**					
Never married	1	1	1	1	1
Separated/divorced	1.42(0.96-2.10)	2.07(0.98-4.38)	0.90(0.51-1.57)	1.34(0.39-4.57)	0.82(0.33-2.03)
Widowed	1.30(0.88-1.90)	1.25(0.62-2.53)	1.13(0.64-1.99)	1.85(0.56-6.06)	0.80(0.33-1.92)
Currently married	1.15(0.76-1.73)	0.91(0.40-2.07)	1.23(0.68-2.22)	2.37(0.70-7.97)	1.18(0.48-2.90)
**Consumption per capita Tertiles**					
T1	1	1	1	1	1
T2	1.49(1.24-1.79) ***	1.22(0.81-1.82)	1.30(1.00-1.68) *	1.70(1.02-2.84) *	2.63(1.61-4.30) ***

T3	1.82(1.49-2.23)	***	1.48(0.92-2.35)		1.44(1.09-1.90)	*	2.61(1.50-4.55)	***	5.28(3.28-8.51)	***
**Distance to nearest clinic**										
Less than 1.5km	1		1		1		1		1	
Between 1.5-3km	1.28(1.04-1.56)	*	1.23(0.80-1.89)		1.19(0.90-1.58)		1.03(0.61-1.76)		1.54(1.06-2.24)	*
More than 3km	0.79(0.66-0.94)	**	0.96(0.65-1.42)		0.74(0.58-0.94)	*	0.74(0.46-1.18)		0.83(0.57-1.22)	
**HH Member Relationship**[1]										
None	1		1		1		1		1	
00001	1.28(0.78-2.10)		1.31(0.46-3.74)		1.11(0.59-2.09)		3.93(0.41-37.68)		1.33(0.47-3.76)	
00010	1.61(0.98-2.63)		1.01(0.42-2.43)		1.54(0.78-3.02)		1.11(0.28-4.38)		1.77(0.61-5.11)	
00111	1.57(0.93-2.66)		2.73(0.85-8.79)		1.11(0.53-2.30)		2.10(0.38-11.74)		1.40(0.42-4.69)	
01000	1.59(1.14-2.22)	**	1.05(0.56-1.97)		1.69(1.08-2.63)	*	1.72(0.67-4.43)		2.01(1.03-3.92)	*
01001	1.52(1.04-2.20)	*	1.45(0.69-3.08)		1.70(1.02-2.82)	*	1.41(0.49-4.05)		1.14(0.50-2.61)	
01101	1.01(0.64-1.60)		1.66(0.49-5.61)		1.28(0.68-2.42)		0.99(0.26-3.77)		1.14(0.38-3.40)	
10000	1.59(0.93-2.70)		2.06(0.67-6.33)		1.83(0.85-3.94)		1.00(0.27-3.76)		0.68(0.21-2.20)	
11000	1.72(1.20-2.47)	**	2.30(1.07-4.92)	*	1.58(0.96-2.58)		0.79(0.29-2.10)		1.76(0.84-3.69)	
11001	1.37(0.95-1.98)		1.84(0.84-4.01)		1.48(0.90-2.44)		0.91(0.34-2.41)		1.07(0.49-2.32)	

*** $p<.001$ ** $p<.01$ * $p<.05$

[1]HH Member Relationship has been encoded with the following relations in order of appearance where a **1** indicates at least one of such a relation in the household, and **0** means no such relation exist in the household: Spouse, Child, Parent, Sibling, Other. For example, 11001 indicate a household with at least a spouse, a child, and other relatives.

Table A5. Awareness Indices

	Overall			Females			Males		
	Mean	*SD*	*95% CI*	*Mean*	*SD*	*95% CI*	*Mean*	*SD*	*95% CI*
**Gender**									
Female	0.667	0.383	(0.651-0.683)	0.667	0.383	(0.651-0.683)			
Male	0.604	0.414	(0.585-0.623)				0.604	0.414	(0.585-0.623)
**Age Group**									
40-49	0.587	0.418	(0.555-0.618)	0.644	0.396	(0.604-0.685)	0.515	0.434	(0.467-0.564)
50-59	0.642	0.395	(0.619-0.665)	0.661	0.384	(0.631-0.690)	0.617	0.409	(0.581-0.654)
60-69	0.628	0.393	(0.605-0.652)	0.646	0.381	(0.615-0.678)	0.609	0.404	(0.575-0.644)
70+	0.675	0.392	(0.653-0.698)	0.706	0.375	(0.677-0.736)	0.637	0.410	(0.602-0.673)
**Years of Education**									
No formal education	0.633	0.399	(0.615-0.651)	0.651	0.390	(0.628-0.674)	0.605	0.412	(0.575-0.635)
Some primary (1-7 years)	0.647	0.394	(0.626-0.667)	0.689	0.370	(0.662-0.716)	0.599	0.415	(0.568-0.631)
Secondary or more (8+ years)	0.638	0.404	(0.610-0.666)	0.672	0.387	(0.632-0.712)	0.608	0.416	(0.569-0.648)
**Employment**									
Not working	0.635	0.403	(0.618-0.652)	0.660	0.387	(0.639-0.681)	0.600	0.422	(0.573-0.628)
Employed (part or full time)	0.613	0.403	(0.581-0.644)	0.656	0.387	(0.611-0.701)	0.577	0.413	(0.533-0.620)
Retired	0.658	0.387	(0.636-0.680)	0.686	0.374	(0.657-0.714)	0.626	0.400	(0.593-0.659)
**Marital status**									
Never married	0.606	0.433	(0.546-0.666)	0.724	0.400	(0.645-0.803)	0.492	0.436	(0.407-0.576)
Separated / divorced	0.653	0.393	(0.620-0.687)	0.692	0.363	(0.652-0.733)	0.597	0.426	(0.540-0.654)
Widowed	0.684	0.384	(0.663-0.705)	0.681	0.383	(0.658-0.704)	0.696	0.389	(0.645-0.747)
Currently married	0.610	0.403	(0.593-0.627)	0.630	0.388	(0.603-0.658)	0.598	0.411	(0.575-0.620)
**Household composition**									
Living alone	0.632	0.413	(0.593-0.671)	0.678	0.396	(0.619-0.736)	0.600	0.423	(0.548-0.652)
Living with 1 other person	0.698	0.376	(0.662-0.734)	0.738	0.347	(0.693-0.783)	0.650	0.403	(0.594-0.707)
Living in 3–6 person household	0.647	0.396	(0.629-0.664)	0.681	0.376	(0.659-0.703)	0.599	0.416	(0.570-0.627)
Living in 7+ person household	0.609	0.403	(0.587-0.631)	0.619	0.397	(0.59o-0.648)	0.597	0.409	(0.563-0.631)
**Consumption per capita tertiles**									
T1	0.590	0.415	(0.567-0.612)	0.617	0.398	(0.589-0.645)	0.551	0.436	(0.515-0.587)
T2	0.651	0.391	(0.63o-0.672)	0.675	0.379	(0.648-0.702)	0.620	0.403	(0.587-0.652)
T3	0.675	0.384	(0.655-0.695)	0.715	0.365	(0.687-0.742)	0.634	0.400	(0.604-0.664)
**Distance to nearest clinic**									
Less than 1.5km	0.639	0.397	(0.620-0.659)	0.655	0.387	(0.630-0.681)	0.620	0.408	(0.591-0.649)
Between 1.5-3km	0.675	0.382	(0.652-0.698)	0.696	0.370	(0.666-0.726)	0.647	0.395	(0.611-0.684)
More than 3km	0.611	0.410	(0.589-0.632)	0.659	0.387	(0.631-0.687)	0.555	0.428	(0.522-0.588)
